# {2,2′-[1,1′-(Ethyl­enedioxy­dinitrilo)diethyl­idyne]di-1-naphtholato}nickel(II)

**DOI:** 10.1107/S1600536809023149

**Published:** 2009-06-20

**Authors:** Wen-Kui Dong, Jian-Chao Wu, Jian Yao, Shang-Sheng Gong, Jun-Feng Tong

**Affiliations:** a School of Chemical and Biological Engineering, Lanzhou Jiaotong University, Lanzhou 730070, People’s Republic of China

## Abstract

In the title complex, [Ni(C_26_H_22_N_2_O_4_)], the Ni^II^ atom has a slight distortion toward tetra­hedral geometry from a square-planar structure, coordinated by two O and two N atoms of the tetra­dentate salen-type bis­oxime 2,2′-[1,1′-(ethyl­enedioxy­dinitrilo)diethyl­idyne]di-1-naphtholate (*L* ^2−^) unit, with a mean deviation of 0.022 Å from the N_2_O_2_ plane. The *N*- and *O*-donor atoms are mutually *cis*. The dihedral angle between two naphthalene systems of the *L* ^2−^ ligand is 67.59 (4)°. The crystal structure is stabilized by inter­molecular C—H⋯O and C—H⋯π inter­actions, which link neighbouring mol­ecules into extended chains along the *b* axis.

## Related literature

For multidentate salen-type compounds in coordination chemistry, see: Akine *et al.* (2005 ▶); Dong *et al.* (2009*a* ▶,*b* ▶); Katsuki (1995 ▶); Ray *et al.* (2003 ▶); Sun *et al.* (2008 ▶). For the isostructural Cu complex, see: Dong *et al.* (2009*c* ▶).
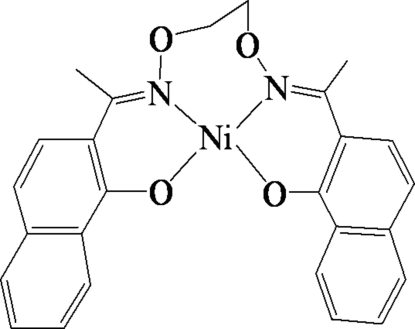


## Experimental

### 

#### Crystal data

[Ni(C_26_H_22_N_2_O_4_)]
*M* *_r_* = 485.17
Monoclinic, 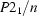

*a* = 13.6975 (13) Å
*b* = 8.2711 (10) Å
*c* = 19.049 (2) Å
β = 95.346 (1)°
*V* = 2148.7 (4) Å^3^
*Z* = 4
Mo *K*α radiation
μ = 0.94 mm^−1^
*T* = 298 K
0.43 × 0.16 × 0.06 mm

#### Data collection

Siemens SMART 1000 CCD diffractometer
Absorption correction: multi-scan (*SADABS*; Sheldrick, 1996 ▶) *T*_min_ = 0.688, *T*_max_ = 0.946
10414 measured reflections
3782 independent reflections
2179 reflections with *I* > 2σ(*I*)
*R*_int_ = 0.073

#### Refinement

*R*[*F* ^2^ > 2σ(*F* ^2^)] = 0.048
*wR*(*F* ^2^) = 0.126
*S* = 1.02
3782 reflections
298 parameters
H-atom parameters constrained
Δρ_max_ = 0.45 e Å^−3^
Δρ_min_ = −0.38 e Å^−3^

### 

Data collection: *SMART* (Siemens, 1996 ▶); cell refinement: *SAINT* (Siemens, 1996 ▶); data reduction: *SAINT*; program(s) used to solve structure: *SHELXS97* (Sheldrick, 2008 ▶); program(s) used to refine structure: *SHELXL97* (Sheldrick, 2008 ▶); molecular graphics: *SHELXTL* (Sheldrick, 2008 ▶); software used to prepare material for publication: *SHELXTL*.

## Supplementary Material

Crystal structure: contains datablocks global, I. DOI: 10.1107/S1600536809023149/hg2525sup1.cif

Structure factors: contains datablocks I. DOI: 10.1107/S1600536809023149/hg2525Isup2.hkl

Additional supplementary materials:  crystallographic information; 3D view; checkCIF report

## Figures and Tables

**Table 1:** Hydrogen-bond geometry (Å, °)

*D*—H⋯*A*	*D*—H	H⋯*A*	*D*⋯*A*	*D*—H⋯*A*
C15—H15*A*⋯O3^i^	0.96	2.45	3.152 (6)	130
C3—H3*C*⋯*Cg*1^ii^	0.97	3.17	4.127 (3)	172
C15—H15*A*⋯*Cg*2^iii^	0.96	3.53	4.398 (3)	152

Symmetry codes: (i) 
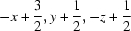
; (ii) 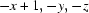; (iii) 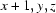.

**Fig. 1.**
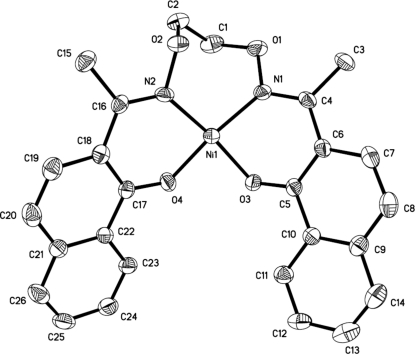
The molecule structure of the title complex. Displacement ellipsoids for non-hydrogen atoms are drawn at the 30% probability level.

**Fig. 2.**
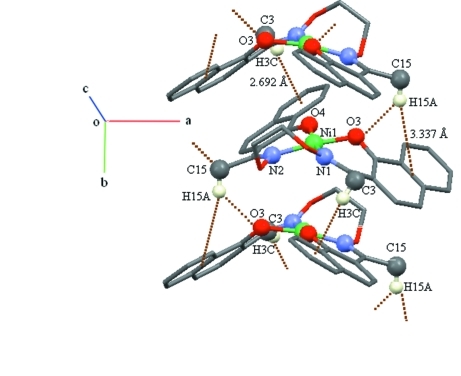
Part of the supramolecular structure of the title complex Showing the formation of C15—H15A···O3 hydrogen bond and C3—H3C···π(benzene), C15—H15A···π(naphthalene) interactions.

## supplementary crystallographic information

### Comment

Multidentate salen-type compounds play an important role in the development of modern coordination chemistry as they readily form stable complexes with most of the transition metals, in which some could exhibit interesting properties (Katsuki, *et al.*, 1995; Akine, *et al.*, 2005; Ray, *et al.*, 2003). Here, we report a new Ni^II^ complex based on the tetradentate salen-type bisoxime ligand 2,2'-[1,1'-ethylenedioxybis(nitriloethylidyne)]dinaphthol (Dong, *et al.*, 2009*a*; Dong, *et al.*, 2009*b*).

In this paper, a new mononuclear nickel(II) complex with salen-type bisoxime chelating ligand, 2,2'-[1,1'-ethylenedioxybis(nitriloethylidyne)]dinaphthol, has been synthesized (Sun, *et al.*, 2008). The dihedral angle between the coordination plane of O3—Ni1—N1 and that of O4—Ni1—N2 is 67.59 (4)°, indicating slight distortion toward tetrahedral geometry from the square planar structure, with a mean deviation of 0.022 Å from the N_2_O_2_ plane. The crystal structure is further stabilized by intermolecular C15—H15A···O3 hydrogen bond and C3—H3C···π(benzene), C15—H15A···π(naphthalene) interactions (Table 1), which link neighbouring molecules into extended chains along the *b* axis.

### Experimental

A solution of nickel(II) chloride tetrahydrate (2.8 mg, 0.0138 mmol) in methanol (3 ml) was added dropwise to a solution of 2,2'-[1,1'-ethylenedioxybis(nitriloethylidyne)]dinaphthol (4.5 mg, 0.0105 mmol) and 99% triethylamine (0.025 ml) in dichloromethane (3 ml). The color of the mixing solution turns to dark-yellow, immediately, and was allowed to stand at room temperature for about three weeks, the solvent was partially evaporated and obtained brown needle-like single crystals suitable for X-ray crystallographic analysis.

### Refinement

Non-H atoms were refined anisotropically. H atoms were treated as riding atoms with distances C—H = 0.96 (CH_3_), C—H = 0.97 (CH_2_), or 0.93 Å (CH), and *U*_iso_(H) = 1.2 *U*_eq_(C) and 1.5 *U*_eq_(O).

### Crystal data

**Table:**

[Ni(C_26_H_22_N_2_O_4_)]	*F*(000) = 1008
*M**_r_* = 485.17	*D*_x_ = 1.500 Mg m^−^^3^
Monoclinic, *P*2_1_/*n*	Mo *K*α radiation, λ = 0.71073 Å
Hall symbol: -P 2yn	Cell parameters from 1885 reflections
*a* = 13.6975 (13) Å	θ = 2.9–22.6°
*b* = 8.2711 (10) Å	µ = 0.94 mm^−^^1^
*c* = 19.049 (2) Å	*T* = 298 K
β = 95.346 (1)°	Prismatic, brown
*V* = 2148.7 (4) Å^3^	0.43 × 0.16 × 0.06 mm
*Z* = 4	

### Data collection

**Table:**

Siemens SMART 1000 CCD area-detector diffractometer	3782 independent reflections
Radiation source: fine-focus sealed tube	2179 reflections with *I* > 2σ(*I*)
graphite	*R*_int_ = 0.073
φ and ω scans	θ_max_ = 25.0°, θ_min_ = 1.8°
Absorption correction: multi-scan (*SADABS*; Sheldrick, 1996)	*h* = −16→15
*T*_min_ = 0.688, *T*_max_ = 0.946	*k* = −9→9
10414 measured reflections	*l* = −22→21

### Refinement

**Table:**

Refinement on *F*^2^	Primary atom site location: structure-invariant direct methods
Least-squares matrix: full	Secondary atom site location: difference Fourier map
*R*[*F*^2^ > 2σ(*F*^2^)] = 0.048	Hydrogen site location: inferred from neighbouring sites
*wR*(*F*^2^) = 0.126	H-atom parameters constrained
*S* = 1.02	*w* = 1/[σ^2^(*F*_o_^2^) + (0.0457*P*)^2^ + 0.4257*P*] where *P* = (*F*_o_^2^ + 2*F*_c_^2^)/3
3782 reflections	(Δ/σ)_max_ < 0.001
298 parameters	Δρ_max_ = 0.45 e Å^−^^3^
0 restraints	Δρ_min_ = −0.38 e Å^−^^3^

### Special details

**Table:**

Geometry. All e.s.d.'s (except the e.s.d. in the dihedral angle between two l.s. planes) are estimated using the full covariance matrix. The cell e.s.d.'s are taken into account individually in the estimation of e.s.d.'s in distances, angles and torsion angles; correlations between e.s.d.'s in cell parameters are only used when they are defined by crystal symmetry. An approximate (isotropic) treatment of cell e.s.d.'s is used for estimating e.s.d.'s involving l.s. planes.
Refinement. Refinement of *F*^2^ against ALL reflections. The weighted *R*-factor *wR* and goodness of fit *S* are based on *F*^2^, conventional *R*-factors *R* are based on *F*, with *F* set to zero for negative *F*^2^. The threshold expression of *F*^2^ > σ(*F*^2^) is used only for calculating *R*-factors(gt) *etc*. and is not relevant to the choice of reflections for refinement. *R*-factors based on *F*^2^ are statistically about twice as large as those based on *F*, and *R*- factors based on ALL data will be even larger.

### Fractional atomic coordinates and isotropic or equivalent isotropic displacement parameters (Å^2^)

**Table:**

	*x*	*y*	*z*	*U*_iso_*/*U*_eq_	
Ni1	0.75831 (4)	0.20469 (8)	0.22591 (3)	0.0404 (2)	
N1	0.7696 (2)	0.2159 (5)	0.12766 (18)	0.0427 (9)	
N2	0.6275 (2)	0.2675 (4)	0.22573 (18)	0.0414 (9)	
O1	0.7023 (2)	0.1391 (4)	0.07716 (16)	0.0612 (10)	
O2	0.5751 (2)	0.2984 (4)	0.15860 (15)	0.0476 (8)	
O3	0.89260 (19)	0.1753 (4)	0.23858 (14)	0.0468 (8)	
O4	0.7640 (2)	0.1617 (4)	0.31931 (15)	0.0513 (9)	
C1	0.6314 (4)	0.0466 (6)	0.1102 (3)	0.0589 (14)	
H1A	0.6614	0.0022	0.1542	0.071*	
H1B	0.6093	−0.0428	0.0798	0.071*	
C2	0.5455 (3)	0.1494 (6)	0.1245 (3)	0.0548 (14)	
H2A	0.5070	0.1728	0.0804	0.066*	
H2B	0.5042	0.0898	0.1542	0.066*	
C3	0.8419 (3)	0.2790 (6)	0.0185 (2)	0.0577 (14)	
H3A	0.8183	0.1769	−0.0002	0.087*	
H3B	0.9075	0.2965	0.0061	0.087*	
H3C	0.8001	0.3640	−0.0010	0.087*	
C4	0.8418 (3)	0.2780 (6)	0.0975 (2)	0.0422 (12)	
C5	0.9487 (3)	0.2779 (5)	0.2089 (2)	0.0390 (11)	
C6	0.9264 (3)	0.3411 (5)	0.1412 (2)	0.0408 (12)	
C7	0.9930 (4)	0.4501 (6)	0.1145 (2)	0.0511 (13)	
H7	0.9782	0.4920	0.0695	0.061*	
C8	1.0781 (4)	0.4966 (6)	0.1518 (3)	0.0561 (14)	
H8	1.1192	0.5704	0.1325	0.067*	
C9	1.1038 (3)	0.4326 (6)	0.2199 (3)	0.0484 (12)	
C10	1.0395 (3)	0.3241 (5)	0.2491 (2)	0.0407 (11)	
C11	1.0631 (3)	0.2647 (6)	0.3181 (2)	0.0497 (13)	
H11	1.0203	0.1940	0.3376	0.060*	
C12	1.1481 (4)	0.3094 (7)	0.3565 (3)	0.0621 (15)	
H12	1.1630	0.2698	0.4019	0.075*	
C13	1.2124 (4)	0.4155 (7)	0.3268 (3)	0.0709 (17)	
H13	1.2707	0.4449	0.3526	0.085*	
C14	1.1914 (4)	0.4762 (6)	0.2610 (3)	0.0653 (15)	
H14	1.2350	0.5474	0.2426	0.078*	
C15	0.4730 (3)	0.3664 (6)	0.2637 (2)	0.0547 (14)	
H15A	0.4715	0.4819	0.2589	0.082*	
H15B	0.4364	0.3351	0.3021	0.082*	
H15C	0.4443	0.3177	0.2209	0.082*	
C16	0.5777 (3)	0.3104 (5)	0.2781 (2)	0.0396 (11)	
C17	0.7106 (3)	0.2156 (6)	0.3666 (2)	0.0415 (11)	
C18	0.6206 (3)	0.2971 (5)	0.3500 (2)	0.0401 (11)	
C19	0.5721 (3)	0.3588 (6)	0.4074 (3)	0.0544 (13)	
H19	0.5140	0.4160	0.3975	0.065*	
C20	0.6067 (4)	0.3380 (6)	0.4755 (3)	0.0588 (14)	
H20	0.5727	0.3823	0.5109	0.071*	
C21	0.6941 (3)	0.2496 (6)	0.4937 (2)	0.0473 (13)	
C22	0.7463 (3)	0.1878 (6)	0.4392 (2)	0.0408 (11)	
C23	0.8325 (3)	0.0997 (6)	0.4558 (2)	0.0508 (13)	
H23	0.8670	0.0590	0.4199	0.061*	
C24	0.8665 (4)	0.0730 (7)	0.5248 (3)	0.0624 (15)	
H24	0.9239	0.0145	0.5354	0.075*	
C25	0.8152 (4)	0.1332 (7)	0.5791 (3)	0.0692 (17)	
H25	0.8382	0.1138	0.6258	0.083*	
C26	0.7319 (4)	0.2199 (7)	0.5639 (3)	0.0620 (15)	
H26	0.6990	0.2607	0.6006	0.074*	

### Atomic displacement parameters (Å^2^)

**Table:**

	*U*^11^	*U*^22^	*U*^33^	*U*^12^	*U*^13^	*U*^23^
Ni1	0.0349 (3)	0.0501 (4)	0.0356 (4)	0.0030 (3)	0.0009 (2)	0.0056 (3)
N1	0.033 (2)	0.056 (3)	0.038 (2)	0.010 (2)	−0.0003 (17)	−0.002 (2)
N2	0.041 (2)	0.046 (3)	0.035 (2)	0.0016 (18)	−0.0066 (17)	0.0077 (18)
O1	0.048 (2)	0.091 (3)	0.043 (2)	−0.004 (2)	−0.0062 (17)	−0.0145 (18)
O2	0.0465 (18)	0.047 (2)	0.0471 (19)	0.0065 (17)	−0.0069 (15)	0.0045 (17)
O3	0.0344 (17)	0.063 (2)	0.0422 (19)	0.0012 (16)	0.0013 (14)	0.0161 (16)
O4	0.0446 (18)	0.072 (3)	0.0380 (19)	0.0152 (17)	0.0097 (15)	0.0141 (16)
C1	0.056 (3)	0.048 (3)	0.069 (4)	0.001 (3)	−0.012 (3)	−0.011 (3)
C2	0.045 (3)	0.057 (4)	0.060 (3)	−0.003 (3)	−0.010 (2)	−0.003 (3)
C3	0.058 (3)	0.083 (4)	0.033 (3)	0.022 (3)	0.005 (2)	0.009 (3)
C4	0.044 (3)	0.052 (3)	0.031 (2)	0.019 (2)	0.004 (2)	0.004 (2)
C5	0.035 (3)	0.042 (3)	0.041 (3)	0.009 (2)	0.008 (2)	0.003 (2)
C6	0.041 (3)	0.043 (3)	0.039 (3)	0.010 (2)	0.007 (2)	0.010 (2)
C7	0.058 (3)	0.054 (4)	0.044 (3)	0.011 (3)	0.015 (3)	0.009 (2)
C8	0.063 (4)	0.043 (3)	0.065 (4)	0.001 (3)	0.023 (3)	0.005 (3)
C9	0.042 (3)	0.046 (3)	0.058 (3)	0.003 (2)	0.009 (3)	−0.004 (3)
C10	0.037 (3)	0.043 (3)	0.042 (3)	0.008 (2)	0.004 (2)	0.000 (2)
C11	0.048 (3)	0.053 (3)	0.047 (3)	0.004 (2)	0.000 (2)	0.002 (2)
C12	0.056 (3)	0.073 (4)	0.053 (3)	0.002 (3)	−0.016 (3)	−0.005 (3)
C13	0.053 (4)	0.065 (4)	0.090 (5)	0.001 (3)	−0.013 (3)	−0.018 (4)
C14	0.054 (3)	0.055 (4)	0.088 (4)	−0.009 (3)	0.012 (3)	−0.003 (3)
C15	0.040 (3)	0.057 (4)	0.067 (3)	0.011 (3)	0.005 (2)	0.006 (3)
C16	0.033 (2)	0.037 (3)	0.049 (3)	−0.002 (2)	0.006 (2)	0.008 (2)
C17	0.038 (3)	0.045 (3)	0.042 (3)	−0.009 (2)	0.006 (2)	0.006 (2)
C18	0.043 (3)	0.032 (3)	0.046 (3)	−0.005 (2)	0.007 (2)	0.005 (2)
C19	0.050 (3)	0.050 (3)	0.065 (4)	0.000 (3)	0.012 (3)	−0.007 (3)
C20	0.066 (4)	0.056 (4)	0.056 (4)	−0.012 (3)	0.019 (3)	−0.018 (3)
C21	0.048 (3)	0.050 (4)	0.044 (3)	−0.018 (2)	0.005 (2)	−0.001 (2)
C22	0.041 (3)	0.046 (3)	0.034 (3)	−0.014 (2)	0.001 (2)	0.010 (2)
C23	0.045 (3)	0.063 (4)	0.044 (3)	−0.011 (3)	−0.002 (2)	0.011 (3)
C24	0.052 (3)	0.080 (4)	0.054 (3)	−0.017 (3)	−0.003 (3)	0.010 (3)
C25	0.071 (4)	0.094 (5)	0.040 (3)	−0.031 (4)	−0.011 (3)	0.007 (3)
C26	0.080 (4)	0.064 (4)	0.043 (3)	−0.030 (3)	0.007 (3)	−0.010 (3)

### Geometric parameters (Å, °)

**Table:**

Ni1—O4	1.809 (3)	C10—C11	1.413 (6)
Ni1—O3	1.849 (3)	C11—C12	1.367 (6)
Ni1—N2	1.865 (3)	C11—H11	0.9300
Ni1—N1	1.894 (3)	C12—C13	1.400 (7)
N1—C4	1.295 (5)	C12—H12	0.9300
N1—O1	1.419 (4)	C13—C14	1.355 (7)
N2—C16	1.309 (5)	C13—H13	0.9300
N2—O2	1.430 (4)	C14—H14	0.9300
O1—C1	1.427 (5)	C15—C16	1.508 (5)
O2—C2	1.434 (5)	C15—H15A	0.9600
O3—C5	1.308 (5)	C15—H15B	0.9600
O4—C17	1.292 (5)	C15—H15C	0.9600
C1—C2	1.498 (6)	C16—C18	1.444 (6)
C1—H1A	0.9700	C17—C18	1.415 (6)
C1—H1B	0.9700	C17—C22	1.442 (6)
C2—H2A	0.9700	C18—C19	1.426 (6)
C2—H2B	0.9700	C19—C20	1.349 (6)
C3—C4	1.506 (5)	C19—H19	0.9300
C3—H3A	0.9600	C20—C21	1.419 (7)
C3—H3B	0.9600	C20—H20	0.9300
C3—H3C	0.9600	C21—C26	1.409 (6)
C4—C6	1.459 (6)	C21—C22	1.410 (6)
C5—C6	1.398 (5)	C22—C23	1.398 (6)
C5—C10	1.449 (6)	C23—C24	1.370 (6)
C6—C7	1.411 (6)	C23—H23	0.9300
C7—C8	1.362 (6)	C24—C25	1.395 (7)
C7—H7	0.9300	C24—H24	0.9300
C8—C9	1.415 (6)	C25—C26	1.356 (7)
C8—H8	0.9300	C25—H25	0.9300
C9—C10	1.407 (6)	C26—H26	0.9300
C9—C14	1.417 (6)		
			
O4—Ni1—O3	83.93 (12)	C9—C10—C5	119.8 (4)
O4—Ni1—N2	90.58 (14)	C11—C10—C5	120.7 (4)
O3—Ni1—N2	168.72 (15)	C12—C11—C10	121.0 (5)
O4—Ni1—N1	168.87 (14)	C12—C11—H11	119.5
O3—Ni1—N1	87.90 (13)	C10—C11—H11	119.5
N2—Ni1—N1	98.73 (14)	C11—C12—C13	119.3 (5)
C4—N1—O1	110.6 (3)	C11—C12—H12	120.3
C4—N1—Ni1	126.4 (3)	C13—C12—H12	120.3
O1—N1—Ni1	122.7 (3)	C14—C13—C12	121.2 (5)
C16—N2—O2	112.3 (3)	C14—C13—H13	119.4
C16—N2—Ni1	130.0 (3)	C12—C13—H13	119.4
O2—N2—Ni1	117.0 (2)	C13—C14—C9	120.8 (5)
N1—O1—C1	111.5 (3)	C13—C14—H14	119.6
N2—O2—C2	110.4 (3)	C9—C14—H14	119.6
C5—O3—Ni1	118.4 (3)	C16—C15—H15A	109.5
C17—O4—Ni1	130.0 (3)	C16—C15—H15B	109.5
O1—C1—C2	110.7 (4)	H15A—C15—H15B	109.5
O1—C1—H1A	109.5	C16—C15—H15C	109.5
C2—C1—H1A	109.5	H15A—C15—H15C	109.5
O1—C1—H1B	109.5	H15B—C15—H15C	109.5
C2—C1—H1B	109.5	N2—C16—C18	120.5 (4)
H1A—C1—H1B	108.1	N2—C16—C15	119.9 (4)
O2—C2—C1	112.1 (4)	C18—C16—C15	119.5 (4)
O2—C2—H2A	109.2	O4—C17—C18	123.1 (4)
C1—C2—H2A	109.2	O4—C17—C22	116.8 (4)
O2—C2—H2B	109.2	C18—C17—C22	120.1 (4)
C1—C2—H2B	109.2	C17—C18—C19	117.2 (4)
H2A—C2—H2B	107.9	C17—C18—C16	121.2 (4)
C4—C3—H3A	109.5	C19—C18—C16	121.5 (4)
C4—C3—H3B	109.5	C20—C19—C18	123.0 (5)
H3A—C3—H3B	109.5	C20—C19—H19	118.5
C4—C3—H3C	109.5	C18—C19—H19	118.5
H3A—C3—H3C	109.5	C19—C20—C21	120.9 (5)
H3B—C3—H3C	109.5	C19—C20—H20	119.5
N1—C4—C6	119.1 (4)	C21—C20—H20	119.5
N1—C4—C3	121.2 (4)	C26—C21—C22	118.0 (5)
C6—C4—C3	119.6 (4)	C26—C21—C20	123.3 (5)
O3—C5—C6	123.6 (4)	C22—C21—C20	118.7 (4)
O3—C5—C10	116.9 (4)	C23—C22—C21	119.8 (4)
C6—C5—C10	119.5 (4)	C23—C22—C17	120.3 (4)
C5—C6—C7	118.5 (4)	C21—C22—C17	119.9 (4)
C5—C6—C4	119.1 (4)	C24—C23—C22	120.3 (5)
C7—C6—C4	121.9 (4)	C24—C23—H23	119.8
C8—C7—C6	122.9 (4)	C22—C23—H23	119.8
C8—C7—H7	118.5	C23—C24—C25	120.3 (5)
C6—C7—H7	118.5	C23—C24—H24	119.9
C7—C8—C9	120.0 (5)	C25—C24—H24	119.9
C7—C8—H8	120.0	C26—C25—C24	120.2 (5)
C9—C8—H8	120.0	C26—C25—H25	119.9
C10—C9—C8	119.2 (4)	C24—C25—H25	119.9
C10—C9—C14	118.3 (5)	C25—C26—C21	121.4 (5)
C8—C9—C14	122.5 (5)	C25—C26—H26	119.3
C9—C10—C11	119.4 (4)	C21—C26—H26	119.3
			
O4—Ni1—N1—C4	−77.6 (9)	C14—C9—C10—C5	179.4 (4)
O3—Ni1—N1—C4	−34.9 (4)	O3—C5—C10—C9	179.2 (4)
N2—Ni1—N1—C4	135.9 (4)	C6—C5—C10—C9	0.4 (6)
O4—Ni1—N1—O1	95.3 (8)	O3—C5—C10—C11	−2.4 (6)
O3—Ni1—N1—O1	138.1 (3)	C6—C5—C10—C11	178.8 (4)
N2—Ni1—N1—O1	−51.1 (3)	C9—C10—C11—C12	−0.8 (7)
O4—Ni1—N2—C16	18.8 (4)	C5—C10—C11—C12	−179.2 (4)
O3—Ni1—N2—C16	−41.9 (9)	C10—C11—C12—C13	−0.1 (7)
N1—Ni1—N2—C16	−167.3 (4)	C11—C12—C13—C14	0.9 (8)
O4—Ni1—N2—O2	−171.9 (3)	C12—C13—C14—C9	−0.7 (8)
O3—Ni1—N2—O2	127.5 (6)	C10—C9—C14—C13	−0.2 (7)
N1—Ni1—N2—O2	2.0 (3)	C8—C9—C14—C13	178.3 (5)
C4—N1—O1—C1	170.0 (4)	O2—N2—C16—C18	−176.5 (4)
Ni1—N1—O1—C1	−3.9 (5)	Ni1—N2—C16—C18	−6.7 (6)
C16—N2—O2—C2	−110.3 (4)	O2—N2—C16—C15	6.5 (6)
Ni1—N2—O2—C2	78.6 (3)	Ni1—N2—C16—C15	176.3 (3)
O4—Ni1—O3—C5	−136.0 (3)	Ni1—O4—C17—C18	14.1 (6)
N2—Ni1—O3—C5	−74.8 (8)	Ni1—O4—C17—C22	−166.5 (3)
N1—Ni1—O3—C5	51.5 (3)	O4—C17—C18—C19	−176.3 (4)
O3—Ni1—O4—C17	147.7 (4)	C22—C17—C18—C19	4.3 (6)
N2—Ni1—O4—C17	−22.5 (4)	O4—C17—C18—C16	6.6 (7)
N1—Ni1—O4—C17	−169.4 (7)	C22—C17—C18—C16	−172.8 (4)
N1—O1—C1—C2	87.7 (4)	N2—C16—C18—C17	−9.8 (7)
N2—O2—C2—C1	−62.0 (5)	C15—C16—C18—C17	167.3 (4)
O1—C1—C2—O2	−48.8 (5)	N2—C16—C18—C19	173.2 (4)
O1—N1—C4—C6	−170.9 (4)	C15—C16—C18—C19	−9.8 (7)
Ni1—N1—C4—C6	2.8 (6)	C17—C18—C19—C20	−2.1 (7)
O1—N1—C4—C3	6.2 (6)	C16—C18—C19—C20	175.0 (4)
Ni1—N1—C4—C3	179.9 (3)	C18—C19—C20—C21	−1.0 (7)
Ni1—O3—C5—C6	−39.8 (5)	C19—C20—C21—C26	−178.0 (4)
Ni1—O3—C5—C10	141.4 (3)	C19—C20—C21—C22	2.0 (7)
O3—C5—C6—C7	−179.5 (4)	C26—C21—C22—C23	0.2 (6)
C10—C5—C6—C7	−0.7 (6)	C20—C21—C22—C23	−179.8 (4)
O3—C5—C6—C4	−6.9 (6)	C26—C21—C22—C17	−179.8 (4)
C10—C5—C6—C4	171.9 (4)	C20—C21—C22—C17	0.2 (6)
N1—C4—C6—C5	26.4 (6)	O4—C17—C22—C23	−2.9 (6)
C3—C4—C6—C5	−150.8 (4)	C18—C17—C22—C23	176.6 (4)
N1—C4—C6—C7	−161.3 (4)	O4—C17—C22—C21	177.1 (4)
C3—C4—C6—C7	21.6 (6)	C18—C17—C22—C21	−3.4 (6)
C5—C6—C7—C8	−0.1 (7)	C21—C22—C23—C24	0.1 (7)
C4—C6—C7—C8	−172.5 (4)	C17—C22—C23—C24	−179.9 (4)
C6—C7—C8—C9	1.3 (7)	C22—C23—C24—C25	0.2 (7)
C7—C8—C9—C10	−1.6 (7)	C23—C24—C25—C26	−0.7 (8)
C7—C8—C9—C14	179.9 (5)	C24—C25—C26—C21	1.0 (8)
C8—C9—C10—C11	−177.6 (4)	C22—C21—C26—C25	−0.7 (7)
C14—C9—C10—C11	1.0 (6)	C20—C21—C26—C25	179.2 (5)
C8—C9—C10—C5	0.8 (6)		

### Hydrogen-bond geometry (Å, °)

**Table:**

*D*—H···*A*	*D*—H	H···*A*	*D*···*A*	*D*—H···*A*
C15—H15A···O3^i^	0.96	2.45	3.152 (6)	130
C3—H3C···Cg1^ii^	0.97	3.17	4.127 (3)	172
C15—H15A···Cg2^iii^	0.96	3.53	4.398 (3)	152

Symmetry codes: (i) −*x*+3/2, *y*+1/2, −*z*+1/2; (ii) −*x*+1, −*y*, −*z*; (iii) *x*+1, *y*, *z*.
